# Pattern and impact of hepatic adverse events encountered during immune checkpoint inhibitors – A territory‐wide cohort study

**DOI:** 10.1002/cam4.3378

**Published:** 2020-08-11

**Authors:** Stephen Lam Chan, Terry Cheuk‐Fung Yip, Vincent Wai‐Sun Wong, Yee‐Kit Tse, Becky Wing‐Yan Yuen, Hester Wing‐Sum Luk, Rashid Nok‐Shun Lui, Henry Lik‐Yuen Chan, Tony Shu‐Kam Mok, Grace Lai‐Hung Wong

**Affiliations:** ^1^ Department of Clinical Oncology The Chinese University of Hong Kong Hong Kong SAR China; ^2^ Institute of Digestive Disease The Chinese University of Hong Kong Hong Kong SAR China; ^3^ Department of Medicine and Therapeutics The Chinese University of Hong Kong Hong Kong SAR China; ^4^ Medical Data Analytics Centre Pathology The Chinese University of Hong Kong Hong Kong SAR China

**Keywords:** hepatic adverse events, immunotherapy, liver cancer, programmed cell death‐1

## Abstract

**Background:**

Immune checkpoint inhibitors (ICIs) are increasingly used in the treatment of cancers. We aimed to evaluate the incidence and prognostic impact of hepatic adverse events (AEs) in a territory‐wide cohort of patients who received ICIs.

**Methods:**

Patients were identified from a territory‐wide database who received ICIs in 2014‐2018. Hepatic AEs were defined as any elevation of liver biochemistries including serum alanine aminotransferase (ALT), aspartate aminotransferase (AST), or total bilirubin levels. Hepatic AEs were graded according to Common Terminology Criteria for Adverse Events (CTCAE) v5.0.

**Results:**

Total of 1480 patients were identified (mean age 60 years, male 65.5%) and the commonest malignancies being lung cancer (39.6%), liver cancer (16.5%), and gastrointestinal cancer (10.0%). Grade 1‐2 and grade 3‐4 hepatic AEs occurred in 41.3% and 14.9% of patients during ICI treatment, respectively. Patients with liver cancer had the highest rate of hepatic AEs (grade 1‐2:54.1%; grade 3‐4:32.8%). Among 711 patients with hepatic AEs, 383 (53.9%) had raised ALT/AST only, and 328 (46.1%) had concomitant raised ALT/AST and bilirubin levels. In the whole cohort, median overall survival of patients without any hepatic AEs, grade 1‐2 and grade 3‐4 hepatic AEs during ICI treatment was 9.0 months, 7.2 months, and 3.3 months (*P* < .001), respectively. Similar results on overall survival were obtained among different types of cancers.

**Conclusions:**

Hepatic AEs occur in more than half of patients receiving ICIs for cancer treatment, with approximately 15% being grade 3‐4 AEs. Occurrence of hepatic AEs is associated with worse prognosis.

## Graphical abstract

Hepatic adverse events are common and may occur in more than half of patients receiving immune checkpoint inhibitors for cancer treatment. Severe hepatic adverse events of grade 3‐4 occur in 15% and are associated with worse prognosis.

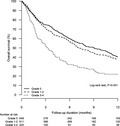

## 1 INTRODUCTION

Immune checkpoint inhibitors (ICIs) have been increasingly used for the treatment of various cancers.^1^, ^2^ During the course of ICIs, derangement of hepatic function is frequently encountered by clinicians. Hepatic adverse events (AEs) in the form of elevation of alanine aminotransferase (ALT)/aspartate transaminase (AST) and/or hyperbilirubinemia are of particular concern to clinicians due to the worry of autoimmune hepatitis.^3^ Aside from autoimmune etiology, hepatic AEs could be due to non‐autoimmune drug‐induced hepatitis, viral hepatitis, alcoholism, biliary obstruction, or progressive malignant disease.^4^ Occurrence of hepatic AEs is theoretically hazardous to patient outcomes due to the delay in the administration of ICIs. Hepatic AEs also signify underlying hepatic injury which may potentially impact the survival of cancer patients.^5^ Currently there have been no dedicated studies on the prognostic impact of hepatic AEs on survival outcomes in the literature.

In phase III clinical trials on the use of ICIs in cancer patients, elevations of liver enzymes were observed in 2 to 10% of patients treated with ICI monotherapy.^6^, ^7^, ^8^ The incidence rate of hepatic AEs is increased to 20%‐30% in populations with hepatocellular carcinoma or treatment with the combination of cytotoxic T‐lymphocyte‐associated protein 4 (CTLA4) antibody and programmed cell death‐1 (PD‐1)/programmed death‐ligand 1 (PD‐L1) antibody.^9^, ^10^, ^11^ A combined use of anti‐angiogenic tyrosine kinase and PD‐1 antibody also increases the incidence of AST/ALT elevation to over 30% (all grade) with approximately 10% of grade 3 or above severity.^12^ The above incidence data are generated from clinical trials on selected tumors. Comprehensive data on overall incidence and pattern of hepatic AEs in real‐world ICI‐treated cancer patients are lacking.

In Hong Kong, ICIs have become available for clinical use since July 2014, and clinicians have commenced using ICIs for the treatment of cancers based on latest clinical trial results. In the current study, we conducted a territory‐wide multicentered cohort study of patients undergoing ICIs to study hepatic AEs. The primary objective was to determine the incidence of hepatic AEs in all cancer patients treated with ICIs. Other objectives include evaluating the pattern of hepatic AEs in different types of malignancies and to study if there are prognostic implications of hepatic AEs. As compared to individual clinical trials, the use of this territory‐wide cohort study has the advantages of having a large sample size, real‐world patients, and the potential to study multiple cancer types simultaneously.^13^

## 2 METHODS

### 2.1 Study design and data source

We performed a retrospective territory‐wide cohort study using data from the Clinical Data Analysis and Reporting System (CDARS) under the management of the Hospital Authority, Hong Kong. CDARS facilitates the retrieval of clinical data captured from different operational systems for analysis and reporting, and provides good quality information to support retrospective clinical and management decisions by integrating the clinical data residing in the data warehouse. It represents inpatient and outpatient data of approximately 80% of the 7.4‐million local population.^14^, ^15^ Patients are de‐identified in CDARS to ensure confidentiality. Clinical data from CDARS have previously been used to conduct different territory‐wide studies.^16^, ^17^, ^18^ The International Classification of Diseases, Ninth Revision, Clinical Modification (ICD‐9‐CM) coding system is used in CDARS; the use of ICD‐9‐CM codes has been found to be 99% accurate based on explicit review of clinical, laboratory, imaging and endoscopy results from the electronic medical records.^19^

### 2.2 Patients

We first identified all consecutive subjects who received at least one dose of ICIs from 1 July 2014 to 31 October 2018 in Hong Kong. We excluded patients without measurement of liver biochemistries at baseline and during follow‐up. Patients were followed from the date of the first prescription of ICIs to the date of death from any cause, censored at date of last follow‐up with liver biochemistries checked before 31 October 2018. A subgroup analysis of patients from the Prince of Wales Hospital, where we have access to their detailed clinical records, was performed. The study protocol was approved by the Joint Chinese University of Hong Kong—New Territories East Cluster Clinical Research Ethics Committee. Informed consent was waived in view of the retrospective nature of this study.

### 2.3 Data collection

Data were retrieved from the CDARS in October 2018. Baseline was defined as the date of first prescription of ICIs. Demographic data including sex and date of birth were captured. At baseline, liver and renal biochemistries, hematological and virological (eg, hepatitis B surface antigen [HBsAg], hepatitis B virus DNA, antibody to hepatitis C virus) parameters were collected (Table S1). Thereafter, serial liver and renal biochemistries were collected until 31 October 2018. Baseline liver biochemistries were defined as those results obtained immediately prior to the first dose of ICIs. We also retrieved data on other relevant diagnoses, procedures, concomitant drugs, and laboratory parameters. We retrieved data on exposure to nucleos(t)ide analogues and (pegylated)‐interferon for patients with hepatitis B (Table S2).

### 2.4 Use of ICIs and indications

The following were the ICIs that had been used in patients: CTLA‐4 antibodies: ipilimumab and tremelimumab; PD‐1 antibodies: nivolumab, pembrolizumab, and spartalizumab; PD‐L1 inhibitors: atezolizumab, avelumab, and durvalumab. These ICIs could be used as monotherapy or in combination. The indications for ICIs were classified according to the type of malignancies treated: lung, gastrointestinal, liver, hematological, and other malignancies (Table S3).

### 2.5 Primary and secondary endpoints

The primary endpoint was hepatic AEs which were defined according to the Common Terminology Criteria for Adverse Events (CTCAE) v5.0,^20^ based on the elevation of ALT and/or AST, or elevation of bilirubin, or both. The upper limit of normal (ULN) of alanine aminotransferase (ALT) and aspartate aminotransferase (AST) was defined according to the criteria of The Asian Pacific Association for the Study of the Liver (40 IU/L for both genders).^21^ The ULN of total bilirubin was defined as 19 µmol/L for both genders. Hepatic AEs occurring during ICI treatment was defined by the period from the commencement of ICIs till 4 weeks after the last dose of ICIs. The secondary endpoint was all‐cause mortality.

### 2.6 Statistical analysis

Data were analyzed using Statistical Product and Service Solutions (SPSS) version 25.0 (SPSS, Inc, Chicago, Illinois), and R software (3.5.3; R Foundation for Statistical Computing, Vienna, Austria). Continuous variables were expressed in mean ± standard deviation or median (interquartile range [IQR]), as appropriate, while categorical variables were presented as frequency (percentage). Qualitative and quantitative differences between subgroups were analyzed by chi‐squared or Fisher's exact tests for categorical parameters and Student's *t* test or Mann‐Whitney test for continuous parameters, as appropriate. Kaplan‐Meier's method was used to estimate overall survival (OS) with 95% confidence intervals (CI); log‐rank test was used to compare the overall survival among patient subgroups. All statistical tests were two‐sided. Statistical significance was taken as *P* < .05.

## 3 RESULTS

### 3.1 Patients’ characteristics

We identified 1509 patients who received ICIs; 29 subjects were excluded due to missing liver biochemistries. Finally, 1480 patients were included and analyzed. At baseline, the mean age was 59.9 ± 13.9 years; 970 (65.5%) were male; 586 (39.6%), 244 (16.5%), 148 (10.0%), 143 (9.7%), and 359 (24.3%) had lung, liver, gastrointestinal, hematological, and other malignancies, respectively (Table 1). The four commonest types of other malignancies included kidney, breast, pharyngeal, and skin malignancies. Among 1480 patients, 1332 (90.0%) received PD‐1/PD‐L1 antibody monotherapy and 145 (9.8%) received combination PD‐1/PD‐L1 and CTLA‐4 antibody treatment. The number of treatment cycles ranged widely from one to 54 cycles, with median (interquartile range) of six (four to nine) cycles. Patients with liver cancer were more likely to be male, positive for HBsAg and antibody to hepatitis C virus, having higher ALT, total bilirubin, and alpha‐fetoprotein, as compared to patients who had non‐liver malignancies (Table 1). Of the 240 patients with positive HBsAg status, 233 (97.1%) received antiviral treatment.

**TABLE 1:** Clinical characteristics of patients at the time of starting immune checkpoint inhibitors

Baseline clinical characteristics	All N = 1,480	Non‐liver cancers N = 1,236	Liver cancer N = 244	*P* value
Male gender (n, %)	970 (65.5)	767 (62.1)	203 (83.2)	<.001
Age (years)	59.9 ± 13.9	60.1 ± 14.0	58.9 ± 12.9	.234
Hemoglobin (g/dL)	11.5 ± 2.1	11.3 ± 2.0	12.3 ± 2.1	<.001
White cell count (x10^9^/L)	7.8 ± 5.4	8.1 ± 5.7	6.2 ± 2.9	<.001
Neutrophil (x10^9^/L)	5.6 ± 4.1	5.8 ± 4.3	4.5 ± 2.7	<.001
Lymphocyte (x10^9^/L)	1.2 ± 1.1	1.3 ± 1.2	1.1 ± 0.5	.001
Eosinophil (x10^9^/L)	0.2 ± 0.3	0.2 ± 0.4	0.2 ± 0.2	.028
Monocyte (x10^9^/L)	0.6 ± 0.4	0.6 ± 0.4	0.5 ± 0.2	<.001
Platelet (x10^9^/L)	254.1 ± 131.9	266.0 ± 132.6	193.5 ± 110.2	<.001
International normalized ratio	1.1 ± 0.2	1.1 ± 0.2	1.2 ± 0.2	<.001
Missing (%)	10.5	11.9	3.3	
Albumin (g/L)	36.2 ± 6.7	36.1 ± 6.7	36.4 ± 6.6	0565
Total bilirubin (μmol/L)	15.1 ± 38.8	11.3 ± 28.3	34.3 ± 68.4	<.001
Alanine aminotransferase (U/L)	23.0 (14.0 ‐ 38.0)	21.0 (13.0 ‐ 33.0)	44.0 (29.0 ‐ 76.0)	<.001
Aspartate aminotransferase (U/L)	30.0 (21.0 ‐ 53.0)	27.0 (20.0 ‐ 38.0)	66.5 (38.0 ‐ 160.8)	<.001
Missing (%)	33.6	38.1	10.7	
Creatinine (μmol/L)	78.7 ± 42.6	77.9 ± 44.6	82.5 ± 30.5	.124
Alpha‐fetoprotein (μg/L)	3.9 (2.4 ‐ 78.8)	2.7 (1.9 ‐ 4.1)	387.1 (13.4 ‐ 6762.5)	<.001
Missing (%)	57.4	68.6	0.4	
Positive HBsAg (n, %)^a^	240 (18.1)	86 (7.6)	154 (77.8)	<.001
Missing (%)	10.3	8.6	18.9	
Positive anti‐HCV (n, %)^a^	13 (2.1)	3 (0.7)	10 (6.2)	<.001
Missing (%)	59.0	64.0	33.6	
Use of ICIs^b^				
PD‐1 Antibody				
Pembrolizumab	838 (56.6)	730 (59.1)	108 (44.3)	<.001
Nivolumab	620 (41.9)	457 (37.0)	163 (66.8)	<.001
Spartalizumab	2 (0.1)	2 (0.2)	0 (0)	1.000
PD‐L1 Antibody				
Atezolizumab	89 (6.0)	89 (7.2)	0 (0)	<.001
Avelumab	3 (0.2)	3 (0.2)	0 (0)	1.000
Durvalumab	3 (0.2)	1 (0.1)	2 (0.8)	.072
CTLA‐4 Antibody				
Ipilimumab	138 (9.3)	70 (5.7)	68 (27.9)	<.001
Tremelimumab	3 (0.2)	0 (0)	3 (1.2)	.004
Type of ICIs				
PD‐1 alone	1,248 (84.3)	1,075 (87.0)	173 (70.9)	<.001
PD‐L1 alone	84 (5.7)	84 (6.8)	0 (0)	
CTLA‐4 alone	3 (0.2)	3 (0.2)	0 (0)	
CTLA‐4 ± PD‐1/PD‐L1	145 (9.8)	74 (6.0)	71 (29.1)	

Note

Alanine aminotransferase, aspartate aminotransferase, alpha‐fetoprotein, and carcinoembryonic antigen were expressed in median (interquartile range), whereas other continuous variables were expressed in mean ± standard deviation. Hypothesis tests compared patients who developed and did not develop liver cancer. Qualitative and quantitative differences between subgroups were analyzed by chi‐squared or Fisher's exact tests for categorical parameters and Student's *t* test or Mann‐Whitney test for continuous parameters, as appropriate.

Abbreviations: anti‐HCV, antibody to hepatitis C virus; HBsAg, hepatitis B surface antigen; ICIs, immune checkpoint inhibitors.

^a^ Percentages were based on non‐missing data.

^b^ One patient may use more than one type of ICIs during follow‐up.

### 3.2 Event

Among 1,480 patients who received ICIs, 831 (56.1%) ever encountered hepatic AEs during the use of ICIs. According to CTCAE v5.0, 428 (28.9%), 183 (12.4%), 167 (11.3%), and 53 (3.6%) had their worst grade of hepatic AEs of grade 1‐4, respectively. Majority of the first hepatic AEs (72.7%) occurred during the between the first and the last dose of ICIs, whereas 27.3% occurred after the last dose of ICIs but within 4 weeks from that. Eight hundred and forty‐three (57.0%) patients died during follow‐up; the median OS from the date of starting ICIs (95% CI) was 6.9 (6.2‐7.7) months, and the median OS from the date of cancer diagnosis (95% CI) was 21.1 (20.0‐22.2) months. In patients with grade 1‐2 hepatic AEs, the median OS (95% CI) from the date of starting ICIs was 8.7 (2.6‐14.7) months in patients with liver cancer and 6.9 (5.8‐8.1) months in patients with non‐liver cancers (log‐rank test, *P* = .089). In patients with grade 3‐4 hepatic AEs, the median OS (95% CI) from the date of starting ICIs was 3.2 (2.4‐3.9) months in patients with liver cancer and 3.4 (2.3‐4.5) months in patients with non‐liver cancers (log‐rank test, *P* = .620).

### 3.3 Comparison of events between patients with liver cancer and non‐liver malignancies

On the one hand, among 244 patients with liver cancer, 212 (86.9%) developed hepatic AEs during the use of ICIs; 63 (25.8%), 69 (28.3%), 60 (24.6%), and 20 (8.2%) had worst severity of grade 1 to 4 hepatic AEs, respectively. On the other hand, among 1,236 patients with non‐liver malignancies, 619 (50.1%) had ever developed hepatic AEs during follow‐up, 365 (29.5%), 114 (9.2%), 107 (8.7%), and 33 (2.7%) had the worst grade of hepatic AEs of grade 1 to 4, respectively. Patients who had liver cancer had more hepatic AEs than patients who had non‐liver malignancies (chi‐squared test, *P* < .001). The median OS (95% CI) from the date of starting ICIs was 6.7 (4.6‐8.8) months in patients with liver cancer and 6.9 (6.1‐7.7) months in patients with non‐liver cancers (log‐rank test, *P* = .708). The median OS from the date of cancer diagnosis (95% CI) was 17.1 (13.0‐21.1) months in patients with liver cancer and 21.7 (20.5‐22.8) months in patients with non‐liver cancers (log‐rank test, *P* = .035).

### 3.4 Rate and pattern of hepatic AEs during ICIs

Table 2 shows the proportion of patients who developed grade 1‐2 and grade 3‐4 hepatic AEs during ICIs (ie, from the commencement of ICIs till 4 weeks after the last dose of ICIs). Grade 1‐2 hepatic AEs during ICIs were common in different cancers. Elevation of ALT and/or AST of grade 1‐2 occurred in 611 (41.3%) patients in the whole cohort (Table 2). Grade 3‐4 hepatic AEs were observed in 220 (14.9%) of the whole cohort with liver cancer contributing a higher proportion (80 out of 220) of these patients. The pattern of hepatic AEs is summarized in Table 3. On the one hand, among 568 patients with elevation of ALT/AST of grade 1‐2, 345 (60.7%) never had concomitant hyperbilirubinemia. On the other hand, among 143 patients with elevation of ALT/AST of grade 3‐4, 17 (11.9%) and 88 (61.5%) of them had concomitant hyperbilirubinemia of grade 1 and grade 2‐4 severity, respectively. Grade 2‐4 hyperbilirubinemia was frequently observed in liver cancers with 54.1% and 81.8% of grade 1‐2 and grade 3‐4 elevation of ALT/AST, respectively (Table 3).

**TABLE 2:** Hepatic adverse events during the use of immune checkpoint inhibitors (ICIs) according to cancer types

	Any hepatic adverse events during ICIs
All cancer (N = 1480)	
Grade 1‐2	611 (41.3)
Grade 3‐4	220 (14.9)
Lung cancer (N = 586)	
Grade 1‐2	208 (35.5)
Grade 3‐4	29 (4.9)
Gastrointestinal cancer (N = 148)	
Grade 1‐2	74 (50.0)
Grade 3‐4	30 (20.3)
Liver cancer (N = 244)	
Grade 1‐2	132 (54.1)
Grade 3‐4	80 (32.8)
Hematological cancer (N = 143)	
Grade 1‐2	73 (51.0)
Grade 3‐4	32 (24.4)
Other cancer (N = 359)^a^	
Grade 1‐2	124 (34.5)
Grade 3‐4	49 (13.6)

Note

All results are presented as n (%) of that cancer type. Hepatic adverse events referred to the worst grade of adverse events occurred during follow‐up. Grade 1 hepatic events referred to ALT and/or AST > 1xULN‐3xULN if baseline was normal; 1.5‐3x baseline if baseline was abnormal, and/or total bilirubin > 1xULN‐1.5xULN if baseline was normal; >1‐1.5x baseline if baseline was abnormal. Grade 2 hepatic events referred to ALT and/or AST > 3xULN‐5xULN if baseline was normal; >3‐5x baseline if baseline was abnormal, and/or total bilirubin > 1.5xULN‐3xULN if baseline was normal; >1.5‐3x baseline if baseline was abnormal. Grade 3 hepatic events referred to ALT and/or AST > 5xULN‐20xULN if baseline was normal; >5‐20x baseline if baseline was abnormal, and/or total bilirubin > 3xULN‐10xULN if baseline was normal; >3‐10x baseline if baseline was abnormal. Grade 4 hepatic events referred to ALT and/or AST > 20xULN if baseline was normal; >20x baseline if baseline was abnormal, and/or total bilirubin > 10xULN if baseline was normal; >10x baseline if baseline was abnormal. During ICIs: this refers to the period from the start of ICIs until 4 weeks after the last dose of ICIs.

Abbreviations: ALT, alanine aminotransferase; AST, aspartate aminotransferase; ICIs, immune checkpoint inhibitors; ULN, upper limit of normal.

^a^ The four commonest types of other cancer included kidney cancer, breast cancer, skin cancer, and pharyngeal cancer.

**TABLE 3:** Pattern of raised alanine aminotransferase (ALT) and/or aspartate aminotransferase (AST), with or without the elevation of total bilirubin (TBili) during the use of immune checkpoint inhibitors (ICIs) according to cancer types

	Any raised ALT and/or AST during ICIs^a^	Raised ALT and/or AST and normal TBili during ICIs^b^	Raised ALT and/or AST and elevated G1 TBili during ICIs^b^	Raised ALT and/or AST and elevated G2‐4 TBili during ICIs^b^
All cancer (N = 1,480)				
Grade 1‐2	568 (38.4)	345 (60.7)	87 (15.3)	136 (23.9)
Grade 3‐4	143 (9.7)	38 (26.6)	17 (11.9)	88 (61.5)
Lung cancer (N = 586)				
Grade 1‐2	183 (31.2)	153 (83.6)	18 (9.8)	12 (6.6)
Grade 3‐4	24 (4.1)	14 (58.3)	3 (12.5)	7 (29.2)
Gastrointestinal cancer (N = 148)				
Grade 1‐2	69 (46.6)	38 (55.1)	9 (13.0)	22 (31.9)
Grade 3‐4	15 (10.1)	2 (13.3)	2 (13.3)	11 (73.3)
Liver cancer (N = 244)				
Grade 1‐2	133 (54.5)	34 (25.6)	27 (20.3)	72 (54.1)
Grade 3‐4	44 (18.0)	2 (4.5)	6 (13.6)	36 (81.8)
Hematological cancer (N = 143)				
Grade 1‐2	67 (46.9)	38 (56.7)	17 (25.4)	12 (17.9)
Grade 3‐4	25 (17.5)	6 (24.0)	2 (8.0)	17 (68.0)
Others (N = 359)^c^				
Grade 1‐2	116 (32.3)	82 (70.7)	16 (13.8)	18 (15.5)
Grade 3‐4	35 (9.7)	14 (40.0)	4 (11.4)	17 (48.6)

Note

Grade 1 raised ALT and/or AST referred to ALT and/or AST > 1xULN‐3xULN if baseline was normal; 1.5‐3x baseline if baseline was abnormal. Grade 2 raised ALT and/or AST referred to ALT and/or AST > 3xULN‐5xULN if baseline was normal; >3‐5x baseline if baseline was abnormal. Grade 3 raised ALT and/or AST referred to ALT and/or AST > 5xULN‐20xULN if baseline was normal; >5‐20x baseline if baseline was abnormal. Grade 4 raised ALT and/or AST referred to ALT and/or AST > 20xULN if baseline was normal; >20x baseline if baseline was abnormal. Grade 1 raised total bilirubin referred to total bilirubin > 1xULN‐1.5xULN if baseline was normal; >1‐1.5x baseline if baseline was abnormal. Grade 2 raised total bilirubin referred to total bilirubin > 1.5xULN‐3xULN if baseline was normal; >1.5‐3x baseline if baseline was abnormal. Grade 3 raised total bilirubin referred to total bilirubin > 3xULN‐10xULN if baseline was normal; >3‐10x baseline if baseline was abnormal. Grade 4 raised total bilirubin referred to total bilirubin > 10xULN if baseline was normal; >10x baseline if baseline was abnormal. During ICIs referred to the period from start of ICIs till 4 weeks after the last dose of ICIs.

Abbreviations: ALT = alanine aminotransferase, AST = aspartate aminotransferase, FU = follow‐up, G1 = grade 1, G2‐4 = grade 2‐4, ICIs = immune checkpoint inhibitors, TBili = total bilirubin, ULN = upper limit of normal.

^a^ Results are presented as N (%) of that cancer type.

^b^ Results are presented as n/N (%). The percentage represents the proportion among patients of that cancer type who had any raised ALT and/or AST during ICIs.

^c^ The four commonest types of other cancers included kidney cancer, breast cancer, skin cancer, and pharyngeal cancer.

### 3.5 Association between hepatic AEs and survival

**FIGURE 1:**
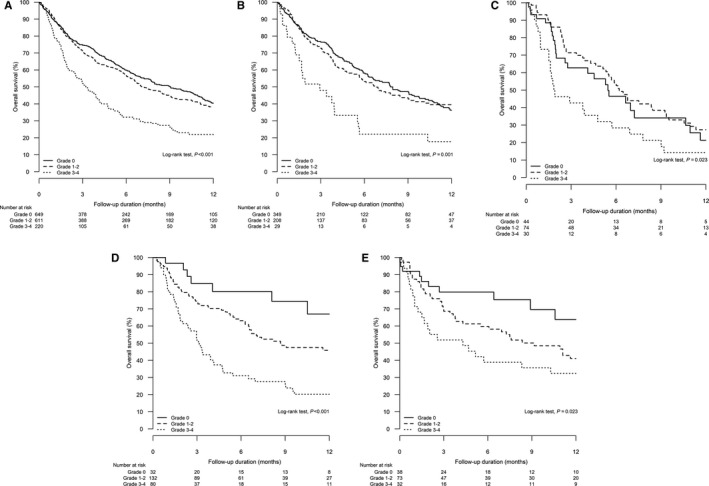
Kaplan‐Meier analysis for overall survival of patients with different severity of hepatic adverse events during the use of immune checkpoint inhibitors (grade 0 *vs* grade 1‐2 *vs* grade 3‐4): A, all patients (log‐rank test, *P* < .001); B, lung cancer (log‐rank test, *P* = .001); C, gastrointestinal cancer (log‐rank test, *P* = .023), D, liver cancer (log‐rank test, *P* < .001), E, hematological cancer (log‐rank test, *P* = .023)

**FIGURE 2:**
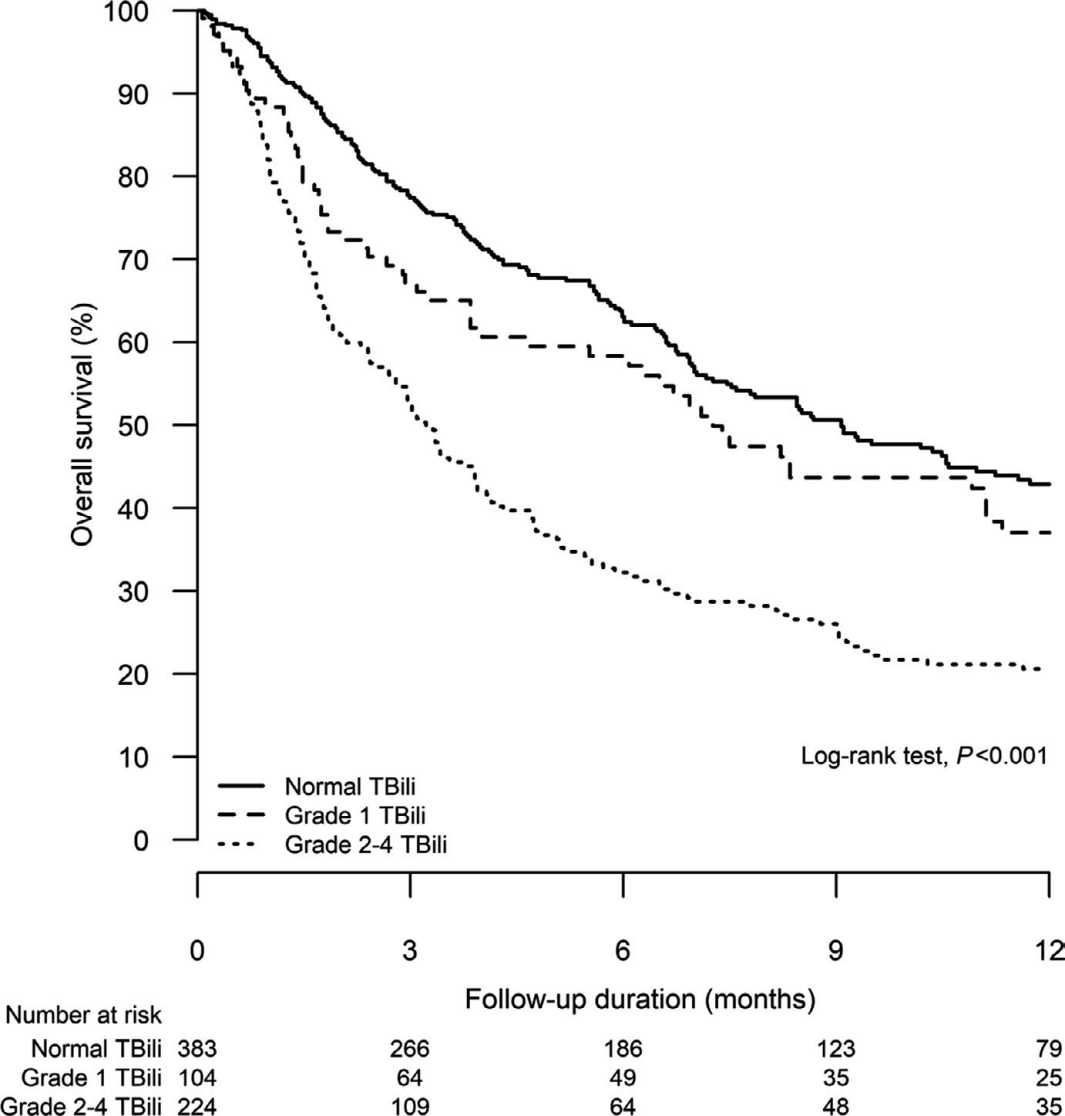
Kaplan‐Meier analysis for overall survival of patients with different severity of elevated total bilirubin (TBili) (grade 0 *vs* grade 1 *vs* grade 2‐4) among all patients with elevated alanine aminotransferase (ALT)/aspartate aminotransferase (AST) (log‐rank test, *P* < .001)

The OS was worse with a higher grade of hepatic AEs. In 1,480 patients, the median OS (95% CI) in patients with absence of, grade 1‐2, and grade 3‐4 hepatic AEs during ICIs was 9.0 (7.3‐10.7) months, 7.2 (6.0‐8.3) months, and 3.3 (2.6‐4.1) months, respectively (log‐rank test, *P* < .001; Figure 1A). Similar results were observed among different types of cancers (Figure 1B‐1E). Among 711 patients with raised ALT/AST, the OS worsened if there was concomitant hyperbilirubinemia. The median OS (95% CI) in patients without, grade 1, and grade 2‐4 elevation of total bilirubin was 9.1 (7.1‐11.1) months, 7.4 (5.6‐9.1) months, and 3.4 (2.6‐4.1) months, respectively (log‐rank test, *P* < .001; Figure 2).

### 3.6 Immune‐related events

Among 962 patients who ever experienced hepatic AEs during follow‐up, 94 (9.7%) of them were administered steroid treatment during a hepatic AE; 66 underwent prednisolone ≥20 mg for more than 1 week and 28 underwent intravenous methylprednisolone treatment. Among steroid users, four and two of them received concurrent mycophenolate mofetil and cyclosporin, respectively, during a hepatic AE. We conducted an independent review of electronic medical records of a subgroup of 252 patients by two specialists in our hospital. Among 252 patients, 142 (56.3%) patients experienced any hepatic AEs during follow‐up; six (4.2%) were immune‐related hepatic AEs. The details of the six patients were described in Table S4.

## 4 DISCUSSION

Real‐world experience of drug use is known to be different from that of clinical trials due to stricter eligibility criteria applied to the latter. For example, studies on renal cell and lung cancers found that over 30%‐70% of treated patients in the real‐world would be ineligible for clinical trials because of their age or aggressive/extensive cancers.^22^, ^23^ In the current study, we report that over half of patients would encounter hepatic AEs during the treatment course of ICIs. This figure is higher than the incidence of hepatic AEs or transaminitis reported by most clinical trials on ICIs. Differences in patients’ characteristics are unlikely to be the sole explanation of this finding because the median age and parameters of hepatic function in the current study population are not remarkably different from that of patients enrolled into trials. However, patients in the current cohort might have more extensive disease and/or be in a later course of their disease, which is evidenced by a median OS of 6.9 months from starting of ICIs and a median OS of 21.1 months from cancer diagnosis. This was a common practice from the period of 2014 to mid‐2018 when ICIs were mostly approved as second‐line or third‐line treatment for cancers by regulatory authorities.^24^ As a result, clinicians tended to reserve ICIs after treatment failures with multiple prior lines of chemotherapy and/or targeted therapies. Under such a background, our study demonstrates a high rate of hepatic AEs during ICIs. The rate of hepatic AEs may become lower in the future when there are more first‐line indications of ICIs which result in their earlier use. Additional studies to evaluate any serial change in incidence of hepatic AEs is warranted in the future.

Among 831 patients with hepatic AEs, approximately 25% (14.9% of the whole cohort) belong to a severity of grade 3‐4 by CTCAE v5.0. This is clinically relevant because the hepatotoxicity of grade 3 or above usually mandate withholding of ICIs and call for additional investigations to evaluate the etiology. The exact cause of hepatic AEs could not be clearly delineated in this database study because of the unavailability of detailed clinical records from other hospitals. To estimate the rate of autoimmune hepatitis, two exploratory analyses were conducted: one by evaluating the rate of protracted use of high‐dose steroids or immunosuppression in the whole cohort; another was by looking into the details of individual patients’ record in our hospital. Based on these methods, the rate of autoimmune hepatitis warranting steroid use was estimated to be 4.2 to 9.7% among patients with hepatic AEs. Nevertheless, this may have underestimated the incidence of autoimmune etiology of hepatic AEs because mild cases of transaminitis could have spontaneously resolved without the use of steroid or immunosuppressants.

Regarding other possible mechanisms leading to hepatic AEs, data show that patients with liver cancers have the highest rate of hepatic AEs of grade 3‐4 toxicity, hence we postulate that progressive liver disease burden would be another important cause of hepatic AEs in these patients.^25^, ^26^ Furthermore, patients with hepatic AEs in liver cancers have the highest chance of concomitant hyperbilirubinemia. This finding suggests that additional biliary obstruction of cholestasis may contribute to the development of hepatic AEs in patients with liver and gastrointestinal cancers. For hematological cancers, it is interesting to note that grade 1‐2 hepatic AEs are frequently encountered in 51% of patients during ICI treatment. The exact cause of hepatic AEs remains unclear: on the one hand, autoimmune hepatitis does not appear to be of significant concern in hematological cancers according to early clinical trials on ICIs in refractory lymphoma with rates of ALT/AST elevation of about 10%.^27^, ^28^, ^29^ On the other hand, the hepatic AEs may be caused by secondary hepatic involvement of systemic lymphoma which has been reported to occur in 16%‐40% of both non‐Hodgkin's and Hodgkin's lymphoma.^30^ Further studies may help validate our observation and elucidate the causes of hepatic AEs.

Our current study demonstrates that the occurrence of hepatic AEs during ICI treatment to be of prognostic impact. This phenomenon is universally observed across all cancer types in this study. Patients with grade 3‐4 hepatic AEs have the worst outcome with median OS of shorter than 4 months. This is predictable because significantly deranged hepatic function, either as a result of liver injury from the treatment or malignant disease involvement, is associated with a high chance of liver failure.^31^ The finding of worse OS in patients with grade 1‐2 severity during ICIs is relatively surprising but clinically relevant. According to international guidelines, grade 2 elevation of ALT/AST warrants withholding of ICIs and close monitoring of hepatic function.^4^, ^32^ After excluding nonimmune causes, corticosteroids are indicated when there is a worsening trend of AST/ALT.^4^, ^32^ Our findings not only support the above guidelines that promulgate aggressive monitoring and management at hepatic AEs of grade 2 severity, but also raise the possibility of considering a lower threshold of initiating investigations and close monitoring even in cases of grade 1 elevations of ALT/AST. Similarly, patients with any elevation of bilirubin, albeit at grade 1 severity, have worse prognoses than those without. This finding is also in accordance with current guidelines that recommend steroid use when rises in ALT/AST are associated with concomitant hyperbilirubinemia of any degree.^4^, ^32^

There are several caveats of the current study. First, this is a retrospective study with conventional limitations of selection and recall bias. These limitations are partly overcome by using a database that covers 80% of patients treated in Hong Kong and the objective nature of the parameters of hepatic function and survival outcomes. Second, detailed data on tumor staging, response to treatment, and causes of hepatic AEs of individual patients are not available in this territory‐wide database study. Hence further analyses on the mechanisms behind the negative prognostic impact of hepatic AEs are not feasible. Third, about 18% of the study population has chronic hepatitis B infection raising the possibility of HBV reactivation during ICI treatment. However, the rate of HBV reactivation is expected to be very low due to the local practice of routine prescription of nucleos(t)ide antivirals during cancer treatment (Supplementary table 2). Recent clinical trial data on ICI use in hepatocellular carcinoma also suggest low rates of HBV reactivation in patients with adequately suppressed viral load. Fourth, we captured the hepatic AEs within 4 weeks after stopping ICIs, in addition to the hepatic AEs occurred during the period of ICI use, because delayed immune‐related events have been reported.^33^ This definition may have led to capturing higher hepatic AEs which are not necessarily due to ICIs. To address this issue, we did a sensitivity analysis by excluding hepatic AEs found during the 4‐week period after cessation of ICI (data not shown), and it was found that results and conclusions were grossly unaltered. Finally, the study population is mainly of Chinese ethnicity hence caution should be exercised when extrapolating results to other regions.

In conclusion, the current study finds that hepatic AEs occur in more than half of the patients receiving ICI immunotherapy, with 15% belonging to grade 3‐4 severity. Patients with liver and gastrointestinal cancers are particularly susceptible to hepatic AEs. Development of hepatic AEs during ICI is associated with a worse prognosis. Clinicians should be vigilant in identifying hepatic AEs and closely monitoring hepatic function during ICI treatment.

## CONFLICT OF INTEREST

Stephen Chan has served as an advisory committee member for MSD, Astra‐Zeneca, Eisai and Ipsen. Terry Yip has served as a speaker for Gilead Sciences. Vincent Wong has served as an advisory committee member for 3V‐BIO, AbbVie, Allergan, Echosens, Gilead Sciences, Janssen, Novartis, Novo Nordisk, Perspectum Diagnostics, Pfizer and Terns; and a speaker for Bristol‐Myers Squibb, Echosens, Gilead Sciences and Merck. He has also received a research grant from Gilead Sciences. Henry Chan has served as an advisory committee member for AbbVie, Aligos, Aptorum, Altimmune, Arbutus, ContraVir, Intellia, Janssen, Gilead, Medimmune, Roche, Vaccitech, VenetoRx, Vir Biotechnology and GRAIL; and as a speaker for AbbVie, Gilead and Roche. Tony Mok has served as a member of the board of directors for AstraZeneca, Chi‐Med, and Sanomics, has received grants or research support from AstraZeneca, Bristol‐Myers Squibb, Clovis Oncology, Merck Sharp & Dohme, Novartis, Pfizer, Roche, SFJ Pharmaceuticals, and XCovery, speakers’ fees from AstraZeneca, Bristol‐Myers Squibb, Boehringer Ingelheim, Eli Lilly, Merck Sharp & Dohme, Novartis, Pfizer, Roche/Genentech, Taiho, and Takeda Oncology, honoraria from ACEA Biosciences, AstraZeneca, Boehringer Ingelheim, Bristol‐Myers Squibb, Celgene, Eli Lilly, Fishawack Facilitate, Ignyta, Janssen, Merck Serono, Merck Sharp & Dohme, Novartis, OncoGenex Pharmaceuticals, Pfizer, Roche/Genentech, SFJ Pharmaceuticals, Takeda Oncology, and Vertex Pharmaceuticals, is a major stockholder in Sanomics, and is an advisory board member for ACEA Biosciences, AstraZeneca, Boehringer Ingelheim, Bristol‐Myers Squibb, Celgene, ChiMed, Cirina, Clovis Oncology, Eli Lilly, Fishawack Facilitate, geneDecode Co, Ignyta, Janssen, Pfizer, Merck Serono, Merck Sharp & Dohme, Novartis, Roche/Genentech, SFJ Pharmaceuticals, Takeda, and Vertex Pharmaceuticals. Grace Wong has served as an advisory committee member for Gilead Sciences and Janssen, and as a speaker for Abbott, Abbvie, Bristol‐Myers Squibb, Echosens, Gilead Sciences, Janssen, and Roche. The other authors declare no competing interests.

## Author Contributions

Stephen Chan, Terry Yip, Cathy Tong, Yee‐Kit Tse, and Grace Wong had full access to all of the data in the study and take responsibility for the integrity of the data and the accuracy of the data analysis. All the authors were responsible for the study concept and design, interpretation of data, the drafting, and critical revision of the manuscript for important intellectual content. Terry Yip, Becky Yuen, Hester Luk, and Grace Wong were responsible for the acquisition and analysis of data.

## Data Availability

De‐identified data may be available upon request made to corresponding author (subject to the approval of local ethic committee).
